# Editorial: Recent Advances in Responsive Optical Nanomaterials

**DOI:** 10.3389/fchem.2021.760187

**Published:** 2021-09-15

**Authors:** Zhiwei Li, Le He, Jingbin Zeng

**Affiliations:** ^1^ Department of Chemistry, University of California, Riverside, Riverside, CA, United States; ^2^ Institute of Functional Nano and Soft Materials (FUNSOM), Jiangsu Key Laboratory for Carbon-Based Functional Materials and Devices, Soochow University, Suzhou, China; ^3^ College of Science, China University of Petroleum (East China), Qingdao, China

**Keywords:** smart and intelligent technologies, responsive materials, optical nanomaterials, colloidal assemblies, biomedical applications, photocatalysis

Responsive optical nanoparticles comprise nanostructured materials with tunable optical properties in response to external stimuli. Due to these unique features, they are often referred to as smart optical nanomaterials (Blum et al., 2015; Li and Yin, 2019). One compelling feature of these remarkable nanostructures is their optical property changes in response to a diverse set of stimuli, including both local environmental changes (temperature, pH, vapors, ionic strength, depletant, humidity, solvent, *etc.*) and remote stimuli (electric field, ultrasound, magnetic field, mechanical force, gravity force, light, X-ray, *etc.*). A few famous materials in this regard include photonic crystals (He et al., 2012; Fenzl et al., 2014), plasmonic nanostructures (Jiang et al., 2017; Zeng et al., 2020), and photo-catalysts (Teixeira et al., 2018). Because of their broad applications in sensing, color display, anticounterfeiting, catalysis, and biomedicine, chemists have developed many working principles and strategies for synthesizing smart nanostructured materials and exploiting their unique optical properties. With these exciting developments, this invited Research Topic covers the synthesis, assembly of smart optical nanomaterials and their emerging applications in biomedicine, sensing, and photocatalysis, which includes one minireview, one review, and five original research articles contributed from 38 researchers.

In developing these attractive optical nanomaterials, synthesizing responsive nanostructures, and assembling them into more complex secondary structures (including both photonic crystals and plasmonic superstructures) are important. The chemical components, shapes, sizes of nanoparticles and periodicity, order, and orientation of superstructure determine the material performances and smart responses upon external stimuli. To this end, plasmonic nanomaterials, particularly noble-metal Au and Ag, have gained great success because of their unique localized surface plasmon resonance (LSPR), which produces sharp extinction peaks and exhibits bright color complementary to the peak position. Previous extensive studies have demonstrated that the plasmonic properties of metal nanostructures are very sensitive to their chemical components, sizes, and shapes. For example, core/shell nanostructures with metal shells have demonstrated superior plasmonic properties, such as widely tunable LSPR peaks and enhanced scattering properties (Li et al., 2020). In this research topic, FePt–Au core-shell nanoparticles are reported by reducing Au precursors at high temperature (Wei et al.). This method produces Au nanoshells with tunable shell thicknesses and optical properties. In addition to these intrinsic particle properties, plasmonic nanostructures can exhibit dynamic color-switching when being assembled into superstructures due to the plasmon coupling between neighboring particles. Their optical properties can be further tuned by controlling interparticle separation upon external stimuli. To this end, Liu et al. created a pH-responsive assembly and disassembly of Au nanoparticles in colloidal dispersions (Liu et al.). In this work, they introduced 3-aminopropyltriethoxysilane (APTES) as the capping ligand and pH-responsive agent to modify Au nanoparticles. The condensation and decomposition of APTES on the Au nanoparticle surface are highly sensitive to the pH of the solution, leading to reversible assembly and disassembly of dispersed Au nanoparticles. This sequence of events produces tunable, pH-sensitive color switching in the Au nanoparticle dispersion. In a minireview article, Lu et al. summarized recent research activities in developing smart plasmonic nanostructures for advanced cancer imaging and treatment (Lu et al.), which covers both the colloidal synthesis of plasmonic nanostructures with controllable sizes and shapes and their self-assembly into superstructures with orientational and/or positional orders. Based on these synthetic strategies, smart theranostic platforms have also been summarized in this minireview for biosensing, background-free imaging, and responsive cancer treatment. Compared with plasmonic nanostructures, photonic crystals exhibit similar color changes in response to external stimuli but through a distinct working mechanism. Specifically, the constructive and deconstructive interference of light creates stopbands in the optical spectrum, producing the unique structural colors and diffraction peaks of photonic crystals. Albeit full of challenges, scientists are leading their research to develop reliable methods to tune the order, periodicity, and phase of photonic crystals for emerging optical properties and responses. Among their various applications, colorimetric sensing and spectroscopic detection is the most attractive one because of the perceptible color changes of photonic crystals. To this end, a review article is delivered to address recent advances in the sensing applications of molecularly imprinted photonic crystals (Fan et al.).

Such remarkable responsive materials are promising in solving existing challenges in various research fields, including cancer treatment, energy conversion, photocatalysis, sensing, anticounterfeiting, and color displays. To showcase their superior performances over conventional materials, three original research articles are included in this research topic, which focuses on photocatalytic degradation of organic dyes upon light irradiation and tumor theranostics based on synergistic nanoplatforms. Yin et al. reported a hydrothermal method to synthesize a photocatalyst, WO_3_, that has high absorptance in ultraviolet and visible spectra (Yin et al.). Utilizing their superior photothermal conversion, this light-active material has exhibited high efficiency in methylene blue degradation. In another work, an amygdaloidal Bi_2_S_3_ nanostructure was reported for enhanced degradation of rhodamine B under near-infrared light irradiation (Yin et al.). In addition to photocatalysis, tumor imaging and treatment also benefit from the creation of smart optical nanomaterials. Shuangchen Ruan and coworkers developed synergistic nanoplatforms for multimodal tumor imaging and therapy triggered by near-infrared light (Wu et al.). In this work, they successfully synthesized sulfide composite nanoflowers, and the systematic studies demonstrated a good photothermal conversion efficiency and a photothermal–chemodynamic–photodynamic synergetic therapeutic effect.

We thank all the researchers who have devoted valuable time and effort to presenting these interesting research and to preparing excellent minireviews, reviews, and original research articles. These valuable summaries of research activities and new progress will inspire the development of emerging smart optical nanomaterials for many fascinating applications.
